# Cognition and Pain: A Review

**DOI:** 10.3389/fpsyg.2021.673962

**Published:** 2021-05-21

**Authors:** Tanvi Khera, Valluvan Rangasamy

**Affiliations:** Department of Anesthesia Critical Care and Pain Medicine, Beth Israel Deaconess Medical Center, Harvard Medical School, Boston, MA, United States

**Keywords:** cognition, pain, memory, cognitive behavior therapy, chronic pain

## Abstract

Cognition is defined as the brain’s ability to acquire, process, store, and retrieve information. Pain has been described as an unpleasant sensory or emotional experience, and for experiencing pain consciously, cognitive processing becomes imperative. Moreover, evaluation of pain strongly depends on cognition as it requires learning and recall of previous experiences. There could be a possible close link between neural systems involved in cognition and pain processing, and studies have reported an association between pain and cognitive impairment. In this narrative review, we explore the available evidence that has investigated cognitive changes associated with pain. We also examine the anatomical, biochemical, and molecular association of pain and neuro-cognition. Additionally, we focus on the cognitive impairment caused by analgesic medications. There is a need to improve our understanding of pathophysiology and cognitive impairment mechanisms associated with chronic pain and its treatment. This area provides a diverse opportunity for grounding future research, aiding institution of timely interventions to prevent chronic pain and associated cognitive decline, ultimately improving patient care.

## Introduction

The multidimensional pain experience is a prevalent complaint in clinical practice and impacts an individual’s physiologic and psychologic states. Pain is classically defined as an unpleasant sensory or emotional experience associated with actual or potential tissue damage (Raja et al., 2020). Thus, pain is a subjective perceptive phenomenon involving cognitive processing rather than a purely sensory phenomenon (Casey and Lorenz, 2000). Cognition involves the acquisition, processing, storage, and retrieval of information by the brain (Lawlor, 2002). Cognition is composed of critical elements such as attention, perception, memory, motor skills, executive functioning, and verbal and language skills (Gellman and Rick Turner, 2013). Cognition is a vital component of the subjective perception of pain requiring cognitive-evaluation, learning, recall of past experiences, and active decision making (Hansen and Streltzer, 2005; Moriarty et al., 2011). The key aspects of learning and memory require attention, which is enhanced by adding an emotional component to the process (Tyng et al., 2017). Multiple cortical and subcortical brain areas are involved in perception, processing, relaying, and pain modulation. Increasing evidence of the close association between neural systems of cognition and pain shows a bi-directional modulatory role.

Observations in the setting of chronic pain (pain persisting longer than 3 months) usually exceed the duration of the noxious stimulus and have deleterious effects on the psychosocial elements of the individual (Hart et al., 2000; Treede et al., 2019; Walankar et al., 2020). Although chronic pain has long been shown to alter cognitive outcomes, emerging studies over the past decade have drawn particular attention to the multi-dimensional effects of pain on various cognitive domains (Nadar et al., 2016). Apart from the psycho-social influences of pain, it can also impact the functional domains and the quality of life in general (Al Mahrouqi et al., 2020). Further, chronic pain’s economic impact costs $635 billion annually in direct medical costs, loss of productivity, and disability programs (Barrett et al., 2020). Thus, evaluating the relationship between cognition and pain is critical to understanding chronic pain syndromes, their associations with comorbidities, and their psychosocial impact for ultimately improving therapeutic targets and patient outcomes.

In this narrative review, we explore the available evidence and summarize the existing literature on the effects of pain on various cognitive changes. We also examine the anatomical, biochemical and molecular association of pain and neuro-cognition. Additionally, we focus on the cognitive impairment caused by analgesic medications. We highlight the need to improve our understanding of the pathophysiology and the mechanisms of cognitive impairment associated with chronic pain and the treatment thereof.

## Anatomy and Neurophysiology of Pain

Understanding the association between neural systems involved in pain and cognition is central to deciphering the relationship between these two entities. At the most basic level, the pain pathway consists of (1) Transduction, (2) Transmission, (3) Modulation, and (4) Perception (Institute of Medicine, and Committee on Pain, Disability, and Chronic Illness Behavior, 1987; Yam et al., 2018).

Noxious stimuli are transduced via a series of specialized nociceptors into a recognizable signal, transmitted through Aδ (touch, temperature), and C fibers (pain). Most sensory fibers would then project to the dorsal root ganglion (DRG), from where the sensory input is transmitted to the central nervous system (CNS; Bourne et al., 2014). In the event of persistent noxious stimulation, there is an upregulation of A-fibers to perceive pain in addition to C fibers; this is responsible for central sensitization leading to hyperalgesia.

Primarily, C fibers contribute to modulation at the level of DRG by regulating the *N*-methyl-D-aspartate (NMDA) receptor configuration and sensitivity (Neumann et al., 1996). An additional modulation of the ascending pain occurs at the spinal cord level through the release of gamma-aminobutyric acid. Functional loss of the lamina II has been implicated in the development of chronic neuropathic pain (Bourne et al., 2014).

The spinothalamic tracts transmitting pain and temperature sensations run along the ventrolateral spinal cord to the ventral posterior nucleus and the central nucleus of the thalamus. Later, the thalamic projections relay to brainstem reticular formation, hypothalamus, and higher cortical centers. These projections to the reticular formation, periaqueductal gray matter (PAG), and the medial thalamic nuclei are important components of motivational and affective domains of pain (Bourne et al., 2014). The thalamic reorganization is an essential pathway for the development of central pain and neuropathic pain. There is an extensive cortical neural circuitry involved in the perception, localization, and modulation of painful stimuli. This network mostly consists of medial and lateral pain systems. The median system consists of the anterior cingulate cortex (ACC), the insular cortices, and the lateral system consisting of primary (SI) and the secondary somatosensory (SII) cortices. Other cortical areas involved in pain perception are the ventrolateral orbital cortex and the motor cortex (Xie et al., 2009).

The descending pain pathway comprises various supraspinal components – the rostral ventromedial medulla (RVM), the dorsolateral pontomesencephalic tegmentum, and the PAG (Bourne et al., 2014). The PAG-RVM-DH (Dorsal horn) pathway is called the descending pain modulatory pathway. In the limbic system, amygdala is associated with the emotional-affective component of pain and modulation thereof. It is activated in response to noxious stimuli, thus lending the amygdala’s central nucleus the name “nociceptive amygdala” (Neugebauer, 2015). The interconnection between the amygdala and the prefrontal cortex, cingulate cortex, basal ganglia, and the cortico-limbic reverberating loops is implicated in chronic pain conditions such as chronic regional pain syndrome (CRPS), visceral hypersensitivity in irritable bowel syndrome (IBS), chronic pelvic pain (Thompson and Neugebauer, 2017). Monoamines, serotonin (5-HT), dopamine (DA), and norepinephrine (NE) help with modulation of the descending pathway by predominantly acting on lamina I and II of the spinal cord. Dysregulated descending modulation plays a vital role in chronic pain conditions (Bourne et al., 2014) Figure 1.

**FIGURE 1:**
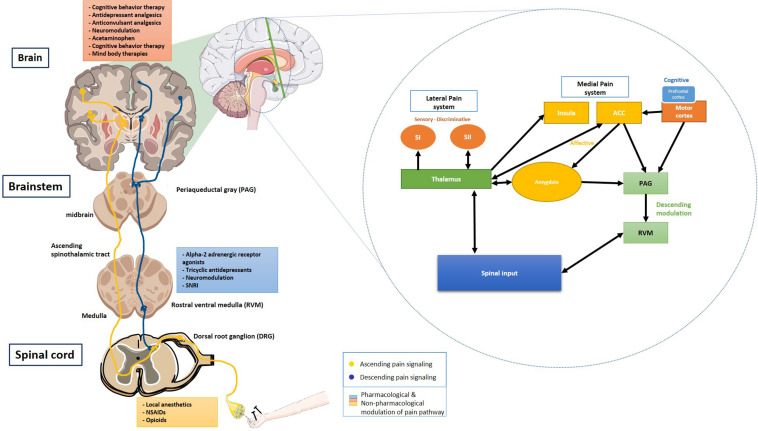
Ascending and descending pain signaling and modulation of pain at each level. Inset shows a schematic of cortical modulation of pain. PAG, Periaqueductal gray; RVM, Rostral ventral medulla; DRG, Dorsal root ganglion; NSAIDs, Non-steroidal anti-inflammatory drugs; SNRI, Serotonin and norepinephrine reuptake inhibitor; SI, Primary somatosensory cortex; and SII, Secondary somatosensory cortex. The schematic art pieces used in this figure were provided by Servier Medical art (http://servier.com/Powerpoint-image-bank). Servier Medical Art by Servier is licensed under a Creative Commons Attribution 3.0 Unported License. Cortical modulation schedmatic adapted from Xie et al. (2009).

## Neuroplasticity and Chronic Pain

Neuroplasticity includes the structural and functional changes that occur in the brain enabling adaptation to environmental cues, learning, memory, and rehabilitation after brain injury (Gulyaeva, 2017). In fact, it is the neurochemical basis of memory formation (Joshi et al., 2019). Neuroplasticity in the context of pain refers to the changes that alter an individual’s response to pain by the development of either chronic pain or hypersensitivity (Basbaum et al., 2009; Gulyaeva, 2017). Neuroplastic adaptations of the brain to chronic pain lead to modulation of cognitive domains, affecting the pain perception.

Imaging studies have suggested a spatiotemporal reorganization of brain activity in relation to chronic pain, during which the representation of pain gradually shifts from sensory to emotional and limbic structures. Thus, the transition of acute pain to chronic pain is a type of activity-induced plasticity of the limbic-cortical circuitry resulting in the reorganization of the neocortex (Thompson and Neugebauer, 2017). Evidence suggests the relationship of the medial prefrontal cortex (mPFC) in the cortico-limbic interaction for modulation of response at the amygdala level. This may offer novel techniques for the control of pain by engaging mPFC control of the amygdala. There is an inter-individual difference in the encoding of painful stimuli and generation of memory for these experiences. This inter-individual difference is based on how the noxious versus the innocuous stimuli are handled and shaped by the individual’s anxiety level. The medial thalamus and ACC are responsible for encoding the stimuli as painful, and the somatosensory cortex discerns non-painful stimuli. This encoding process is also associated with enhanced functional connectivity between the thalamus and the mPFC and is essential to the affective component of pain perception and memory formation (Tseng et al., 2017).

Various neurotrophic factors, neuro-modulatory lipids, and neuropeptides are implicated in the development of plasticity (Duric and McCarson, 2006; Doan et al., 2015). Neuropeptides such as NE, DA, and 5-HT are essential in modulating descending pain signals and the affective component of pain, which is often linked to depression. Similarly, brain-derived neurotrophic factors (BDNF) are associated with the development of synaptic plasticity and *de novo* neurogenesis throughout the peripheral and central pain circuits. Decreased BDNF levels at the hippocampus are found in chronic pain conditions, and this finding is positively linked to the development of depression-like symptoms (Duric and McCarson, 2006; Doan et al., 2015).

A classic example of neuroplasticity in chronic pain setting is neuropathic phantom limb pain (Ramachandran and Rogers-Ramachandran, 2000). Its central pathophysiology involves the complex interaction of cortical elements, memory, and pain perception. Modalities such as non-invasive brain stimulation (NIBS) and cognitive-behavioral therapies (CBT) have shown positive effects in the treatment of neuropathic pain, reinforcing the role of cognition, and cortical perception in the pathophysiology of pain (Kikkert et al., 2019).

Repetitive noxious stimuli often lead to the development of a maladaptive change at the CNS level. This maladaptive change, called the “wind-up phenomenon” or central sensitization, is responsible for developing chronic intractable pain or non-responsive pain (Müller, 2000). This manifests either as hyperalgesia or allodynia. Hyperalgesia, where mildly noxious stimuli are perceived as painful due to resetting of the peripheral nerve threshold. Whereas, in allodynia, there is a recruitment of nerves that carry non-noxious impulses to pain-sensing neurons. Thus, a non-noxious stimulus is perceived as a noxious stimulus in allodynia. Additionally, the spontaneous firing of the DRG may add to the wind-up phenomenon (Gottin et al., 1995; Wilder-Smith, 1995). When sensory stimuli act on modified central neural mechanisms, the output is influenced by the memory of these painful stimuli (Melzack et al., 2006). The pre-emptive analgesia concept focuses on preventing the wind-up phenomenon. This is often achieved by blocking the peripheral transmission of pain by local anesthetics and central perception by using opioids and NMDA receptor antagonists (Gottin et al., 1995; Müller, 2000). There is growing interest in the use of preemptive analgesics in the surgical context. However, various authors have conflicting opinions about the use of preemptive analgesics for surgical patients (Gottin et al., 1995; Wilder-Smith, 1995).

## Clinical Evidence for Pain and Cognition

**FIGURE 2:**
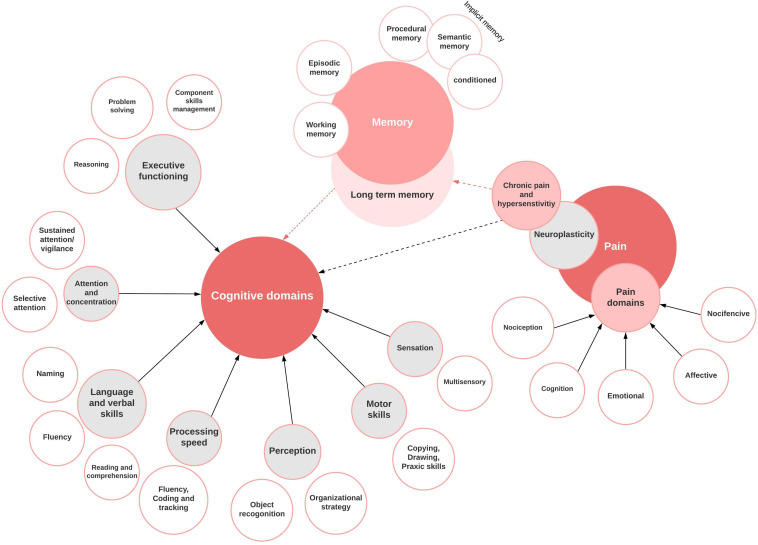
Cognitive domains and the interconnectivity to pain perception and memory formation.

A bi-directional relationship exists between cognition and pain (Villemure and Bushnell, 2002). Disruption of cognitive processing has been investigated in various common chronic pain syndromes, with studies focusing on several cognitive output types. Fibromyalgia, migraine, chronic back pain, rheumatoid arthritis, diabetic neuropathy, osteoarthritis, CRPS, peripheral neuropathic pain syndromes, and multiple sclerosis have been the focus of most clinical studies (Calandre et al., 2002; Dick and Rashiq, 2007; Cousins et al., 2015; Gil-Gouveia et al., 2015; Curatolo et al., 2017; Huang et al., 2017; Jensen et al., 2018; Martinsen et al., 2018; Alemanno et al., 2019; Said et al., 2019; Oláh et al., 2020). Major cognitive parameters investigated encompass attention, learning, memory, sustained concentration, processing speed, psychomotor ability, and executive function Figure 2.

The methodologies employed in these studies comprise a battery of tests, which typically include pain questionnaires such as numerical rating scales, visual analog scales, or McGill pain questionnaire to measure pain, coupled with tests of cognition. Cognitive function may be assessed using subjective self-report measures or objectively with formal, empirically validated neuropsychological tests focusing on one or more aspects of cognition. To provide objectivity to the diagnosis, predicting therapeutic benefits of individualized interventions for chronic pain, the use of fMRI signatures is being investigated. This neural circuit potentially includes the thalamus, anterior and posterior cingulate cortex, and PAG (Wager et al., 2013). Analyzing the change in these signatures over time and with treatment can help pave way for personalized medicine in the future (Thorp et al., 2018). Comorbid affective disorders (such as depression and anxiety) and the effects of sleep disturbance and medication use are sometimes, but not always, considered, and they present an interesting dichotomy in the experimental approach.

### Attention

Attention is the individual’s capacity to process information and involves focused or directed consciousness (Mirsky et al., 1991). A diffuse system of interconnected neurons controls attention at the most fundamental level called the attention matrix. This system receives intrinsic and extrinsic sensory input continually, ultimately impacting the functional adaptations. Attention is a function of cortical and subcortical gray matter structures globally, with interconnected white matter tracts. The frontal lobe and inputs from a widespread network of thalamic and bihemispheric structures are the essential component of the attention neural networks (Filley, 2002). There is an overlap between the pain pathways and attention matrix, which makes the study of attention in pain and the modulation particularly interesting. Legrain et al. (2009) suggested that continued painful stimuli impact attentional control mechanisms required to remove task-irrelevant stimuli, resulting in decreased task performance. In experimental studies, attention is evaluated based on attention span, attention switching between ≥2 cognitive tasks, and divided attention, which studies the ability to process >1 source of information simultaneously (Moore et al., 2019).

Young adults and middle-aged adults can use cognitively demanding tasks to diverge their attention and self-manage pain to some extent (Valet et al., 2004). Pain sensitivity is decreased with engagement in attention requiring tasks and with the use of environmental distractors (Sloan and Hollins, 2017; Hoegh et al., 2019). However, older adults are limited in this capacity, and chronic pain can impair independent living, a risk factor for physical disability, hospitalization, and death (van der Leeuw et al., 2018). Comorbidities such as depression and anxiety can impact both the perception of pain and attention (Shuchang et al., 2011). Thus, chronic pain patients, especially in the older age group with coexisting conditions, require special care in the clinical setting.

Clinical studies over the past decade have shown that there is an increased incidence of patient-reported attention deficits with chronic pain (McCracken and Iverson, 2001; Muñoz and Esteve, 2005; van der Leeuw et al., 2018). The majority of the studies found no association between age, gender, pain-chronicity, anxiety, depression, medication, site of pain, and cognitive performance (Dick and Rashiq, 2007; Martinsen et al., 2018). Interestingly, recent studies show decreased accuracy on attentional switching and divided attention tasks in patients with fibromyalgia (Moore et al., 2019). Animal models used to study the impact of pain on attention and learned behavior (operant nose poke tests) shows increased omissions and decreased accuracy in experimentally induced inflammatory pain (Boyette-Davis et al., 2008; Pais-Vieira et al., 2009). Due to heterogeneity in the chronic pain syndrome type, pain scales used, and cognitive tests for assessment of attention, it is difficult to draw definitive conclusions (Emerson et al., 2020; Rischer et al., 2020).

### Memory

Conceptually, memory consists of a succession of storage systems essential for information flow from the environment to a short-term memory store, which then feeds long-term memory. The information about the environmental cues passes temporary sensory buffers en-route, which are essentially part of perceptual processes (Baddeley, 2010). Working memory is a subset of memory which controls this flow of information in and out of the long-term memory, thus playing an essential role in learning and cognition (Shiffrin, 1977). The hippocampus is associated with long-term explicit memory formation and handling emotional stressors (Zaletel et al., 2016; Sawangjit et al., 2018). Human and animal studies show a decreased hippocampal volume and structural and biochemical plasticity in the setting of chronic pain (Johnston et al., 2012; Mutso et al., 2012; Tajerian et al., 2018). Amygdala is another critical brain region for learning. The basolateral amygdala is activated in the presence of glucocorticoid, which impacts memory consolidation. Furthermore, working memory performance and retrieval are impaired with high glucocorticoid levels (Roozendaal et al., 2006). Involvement of the amygdala in chronic pain has now been shown in human studies and animal models for chronic pain, eliciting the influence of dorsal horn (DH) neuron excitation and the interaction between the amygdala and the mPFC (Neugebauer et al., 2004; Ji et al., 2010).

Studies over the past have demonstrated that chronic pain adversely affects working memory, recall, and recognition memory (McCracken and Iverson, 2001; Muñoz and Esteve, 2005; Berryman et al., 2013). Most patients with chronic pain report poor memory, recall, and concentration in their daily activities (Dufton, 1989; Iezzi et al., 1999, 2004; McGuire, 2013). Interestingly, implicit memory (semantic, procedural, and conditioned) is less likely to be affected by pain owing to its automated nature (Grisart and Van der Linden, 2001). Additionally, most studies concluded that patients with pain have deficits in general and specialized cognitive screening measures (Povedano et al., 2007; Rodríguez-Andreu et al., 2009). It is still unclear whether the intensity and nature of chronic pain syndrome impact semantic memory, immediate or delayed verbal memory, recognition memory, visuospatial memory, and long-term memory (Ryan, 2005; Weiner et al., 2006; Lee et al., 2010). Several groups have modeled pain-related cognitive impairment in animal models and demonstrated poor performance on delayed non-matching to position lever press tasks and delayed novel object recognition (Lindner et al., 1999; Millecamps et al., 2004; Hu et al., 2010). They describe this decreased accuracy, increased response latency for cognitively challenging tasks as the negative effects of pain on spatial learning, recognition, and memory (Hu et al., 2010). Neuropathic rat models are being analyzed to investigate the role of novel molecular therapeutic targets for chronic pain (Qian et al., 2019).

### Processing, Executive Functioning, and Decision Making

Executive function is a set of neurological processes that assist with complex cognitive functions such as planning, organization, thought control, self-regulation, goal-directed actions, initiation, and analyses of actions (Moriarty et al., 2011). Emotional decision-making requires higher executive functioning (Tyng et al., 2017). Anatomically, executive functioning is a higher mental function regulated by the frontal lobes, including the dorsolateral prefrontal cortex (DLPC), ACC, orbitofrontal cortex (Verdejo-García et al., 2006). There is a functional overlap in the pathways in the brain responsible for executive functioning and pain perception. Gray matter reduction is implicated in age-related cognitive decline and an impaired executive functioning and decreased processing speed (Minkova et al., 2017). These age-related gray matter changes are expedited in chronic pain syndromes. ACC, IC, and the DLPC are decreased in volume in patients with chronic pain (Ceko et al., 2013; Lai et al., 2020; Planchuelo-Gómez et al., 2020). Since these areas are also part of the pain neuromatrix, loss of these areas correlates with changes in cognitive executive functioning, and processing speed.

Perception, processing speed, executive functioning, and decision making are studied in patients with chronic pain and have demonstrated that pain adversely affects perceptual learning and emotional decision making (Grisart and Van der Linden, 2001; Apkarian et al., 2004; Barnhart et al., 2019). Patients with pain often show greater harm avoidance, but there is no impact on overall cognition in this subset (Verdejo-García et al., 2009). The type of chronic pain defines the impact on executive functioning. While it is impacted in FM, no correlation is noted in neuropathies and chronic musculoskeletal pain (Grisart and Van der Linden, 2001; Verdejo-García et al., 2009). Interestingly, emotional decision-making is more significantly impacted in patients with chronic lower back pain than CPRS (Apkarian et al., 2004). It is still unclear whether the intensity and nature of the chronic pain syndrome impacts processing speed and attentional interference tasks. Animal models for inflammatory pain show impaired emotional decision-making on rodent gambling tasks (Pais-Vieira et al., 2009; Ji et al., 2010).

### Psychomotor Efficiency and Reaction Time

Clinical studies in patients with chronic pain such as neuropathies have demonstrated that pain adversely impacts performance and psychomotor efficiency, and verbal reaction time (Ryan et al., 1993; Antepohl et al., 2003; Ryan, 2005; Shuchang et al., 2011). The number of pain sites and neuropathy duration is also positively correlated to psychomotor efficiency (Ryan, 2005). It is still unclear whether the intensity and nature of chronic pain syndrome impact motor skills latency and amplitude. The measure of psychomotor efficiency has been most extensively studied and reported in patients with neuropathies, making the generalizability of this observation over different pain cohorts difficult.

## Therapies for Chronic Pain and Impact on Cognition

### Pharmacological Therapies

Management of chronic pain remains a challenge for healthcare professionals. Apart from the treatment of inflammation using non-steroidal anti-inflammatory drugs, routinely used modalities for pain control target the sensory component of pain. The targets comprise of control of pain transmission [opioids and tricyclic antidepressants (TCAs)] and modulation of neuronal excitability (opioids, anticonvulsants) Figure 1.

Opioid receptors are uniquely present at all the neural loci associated with pain signaling and perception (Corder et al., 2018). The opioid system is well-positioned in the brain network to modify the perception of pain. This includes somatosensory neurons of the DRG, excitatory interneurons, and lamina I neurons that relay information to the thalamus and the PAG. The non-linearity of the intensity of the painful stimulus and the perception of pain result from the neural input from the sensory, emotional, interoceptive, inferential, and cognitive information. In the descending pain pathway, opioids act on the PAG, RVM, and spinal cord to modify the nociceptive input perception. Opioid analgesics also act at the level of the rostral, subcortical, and cortical sites, causing an analgesic impact by altering the body’s affective and somatic responses (Corder et al., 2018).

Opioids have unique problems, including constipation, diarrhea, sedation, nausea, vomiting, and pruritus (Benyamin et al., 2008; Pask et al., 2020). Having said that, opioids remain the most preferred analgesic in the setting of chronic pain because of their high potency (Portenoy, 2011). Often patients on long-term therapy develop tolerance to the opioid medication, and higher doses are required to achieve the same therapeutic benefit (Anand et al., 2010). However, higher mean opioid consumption worsens distinct cognitive domains, in particular attention, language, orientation, and psychomotor function. Periodically follow-up with patients on chronic pain medications is essential to diagnose subtle cognition changes (Pask et al., 2020). That said, the critical prespecified adverse events related to opioids, including addiction, depression, and particularly cognitive decline, are often not reported (Els et al., 2017). Evidence suggests that children born to opioid-dependent mothers tend to have a higher incidence of developing cognitive dysfunction, psychomotor disturbance, attention problems, and overall lower IQ as they grow up (Lee et al., 2020). Opioid-induced decreases in arousal are caused by blockage of cholinergic arousal projections from the brainstem to the thalamus and the cortex (Brown et al., 2018). Recent studies show a decrease in the gray matter volume and bilateral amygdalar modulation even with once-daily administration of morphine for 1 month (Lin et al., 2016). Mu opioid receptor and kappa-opioid receptor agonists have been shown to affect normal cognitive function; there is increased psychomotor retardation, decreased accuracy, and impaired recall. This bidirectional impact of pain and cognition has allowed researchers to test opioid antagonists as a potential cognitive-enhancing drug (Jacobson et al., 2018).

Although opioids, TCAs, and anticonvulsant therapies inherently impact the cognitive domains, this impact is inconsistently observed over human and animal studies. The majority of human studies suggest a decrease in attention, processing, memory encoding and retrieval, reaction time, and psychomotor performance with the use of opioids, TCAs, and anticonvulsants (Hindmarch et al., 2005; Sjøgren et al., 2005; Cherrier et al., 2009; Salinsky et al., 2010). However, evidence from some human studies and animal models is equivocal (Jamison et al., 2003; Tassain et al., 2003; Shannon and Love, 2004, 2005). Thus, analgesics in chronic pain and cognition studies can be a potential confounder that cannot be effectively controlled due to ethical reasons.

Endocannabinoids (ECs) are the in-built antinociceptive system of the body. Current research focuses on understanding this EC system better and maximizing its potential to provide safer pain control. The major areas of interest include – EC metabolism inhibition, Anandamide (AEA), and 1-arachidonoyl glycerol (2-AG) are the earliest recognized EC ligands in the body (Devane et al., 1992; Stella et al., 1997; Anand et al., 2010). ECs, AEA, and 2-AG, along with their enzyme modulators, fatty acid amide hydrolase and monoacylglycerol lipase, are essential components for modulation of pain perception and target of most therapeutic interventions. Even though preclinical evidence suggests the role of opioid transmitters in causing cognitive decline, there is not enough evidence to indicate the role of ECs as a potential to decrease cognitive abilities (Woodhams et al., 2017). There is a growing interest in exploring the benefit of cannabis-derived products for managing chronic persistent pain (Wallace et al., 2015). However, it has been shown in recent studies that cannabis products increase the risk of depression and anxiety in adults experiencing chronic pain (Wildes et al., 2020). Thus, individualizing the analgesic therapy (type and dose) based on the patient’s profile is essential for preventing pain and pain-related cognitive decline.

### Non- pharmacological Therapies

Opioids are potent analgesics, however, considering their unique side effect profile and evidence of an emerging opioid epidemic, alternative non-pharmacological therapies are now being explored. Sensory processing of signals can be modulated by altering the attention component of cognitive processing (Petrovic et al., 2000). Modulation of the central sensory processing was successfully investigated by measuring regional cerebral blood flow with Positron Emission Tomography (PET) based technique during cognitively challenging tasks and with a painful stimulus (Petrovic et al., 2000). Neuroimaging studies PET and fMRI have shown a decreased pain perception with simple distraction techniques. This finding points toward the phenomenon of cortical suppression of pain in the presence of a cognitively challenging task. There were an observable increased signal intensity and activation of the cingulo-frontal cortex, including the orbitofrontal and perigenual ACC, as well as PAG and the posterior thalamus (Valet, 2004). This phenomenon is being translated into clinical practice by using innovative techniques such as virtual reality (VR) and augmented reality (AR) as a clinical tool for the management of pain (Pozeg et al., 2017; Pourmand et al., 2018; Chuan et al., 2020).

Cognitive behavior therapy refers to the psychotherapeutic techniques used to decrease pain perception. Traditionally, it includes relaxation therapy, activity pacing, sleep hygiene, scheduling pleasant activity, identifying and modifying cognitive distortions (Barrett et al., 2020). CBT has also shown promise in preventing acute pain conversion to chronic pain (Glare et al., 2020). Pain catastrophizing is a response style that impacts the outcomes of chronic pain patients. Pain catastrophizing is a maladaptive change, and patients often view pain as uncontrollable, permanent, and destructive. CBT is a vital treatment modality for this class of chronic pain patients (Day et al., 2020; Gilliam et al., 2020). Evidence in fibromyalgia patients suggests the clinical benefits of CBT in reducing pain catastrophizing (Lazaridou et al., 2017). Newer CBT methods, such as dialectic pain management (DPM), are being employed to improve and respond to chronic pain therapy (Barrett et al., 2020). DPM skill group sessions include a dialectic integration of change with acceptance to the present moment; this construct is further strengthened with mindfulness practices. Emotions, vulnerabilities, self-compassion, motivation, invalidation, and interpersonal effectiveness, and other personal challenges are addressed that commonly impact chronic pain patients (Barrett et al., 2020). Not just chronic pain, but therapies like acupuncture, hypnosis, mindfulness, relaxation, VR, and AR-based delivery system are being utilized for inpatient and outpatient acute pain such as headache, migraine, and pain in acute and emergency setting (Lindner et al., 2020; Vekhter et al., 2020). As a complementary procedure, acupuncture provides therapeutic benefits by increasing pain thresholds and insular activation (Cao et al., 2019).

Mindfulness meditation and mindfulness-based cognitive therapy have shown therapeutic benefit and improved sensitivity to opioid-based treatment in patients with chronic lower back pain (Zgierska et al., 2016; Day et al., 2019, 2020). Mindfulness-based practices focus on altering the pain catastrophizing, which is an essential component of non-pharmacological therapies for chronic pain. It alters the cognitive content, processing, and negative affectivity, thus leading to a shift critical for any treatment efficacy (Day et al., 2020). Other theoretical models hypothesize that mindful meditation can restructure the pain-related cognitive content, making adaptive changes necessary to counter pain perception. Studies have shown an increase in the EC levels even after short-term meditation practice, providing evidence of the analgesic potential of mind-body therapies (Sadhasivam et al., 2020). Understanding and individualizing the treatment and matching it to the patient’s requirement can improve treatment response, adherence, and outcomes (Day et al., 2020; Zetterqvist et al., 2020). Considering at-home practices and online modules can improve reach and compliance with mindfulness-based therapies (Day et al., 2020; Zetterqvist et al., 2020).

## Future Directions

Clinical and preclinical studies indicate a definitive link between pain and cognitive domains. However, the precise underlying psychological and neural mechanisms, the cognitive deficit associated with each chronic pain condition, and the role of subjective factors, the nature and the duration of the pain are yet to be elucidated. Chronic pain also results in adaptations and alterations in cognitive strategies, further adding to the heterogeneity in interpreting the primary deficit. Neuroplasticity-based reorganizational changes have an essential role in pain permanence and pain modulation and should be considered during the interpretation of the findings.

Lack of consistent cognitive effects across studies, methods, and pain conditions highlight the need for more standardized evaluation methods to allow comparisons to identify global and precise cognitive deficits. The use of different batteries of neurocognitive tests and pain questionnaires prevent a head-on comparison between different chronic pain conditions and their impact on the cognitive domains. The tests should have the sensitivity to identify the intensity of pain and the effect of pain on various cognitive domains. These limitations and the complex nature of the interconnection between pain and cognitive neuro-matrix makes evaluative conclusions rather difficult. Future studies should be designed to address these issues. Furthermore, current research lacks evidence to draw inference for longer-term impacts on cognition in patients with chronic pain. Chronic pain-based animal models have been used to understand the implication of pain on long term cognitive domains; however, these models are limited in their ability to mimic the motivation-affective and evaluative aspects of pain.

Future studies should be modified to incorporate psychophysiological, psychophysical, pharmacological, and brain imaging techniques to evaluate cognitive effects in the setting of chronic pain. Such studies would provide a multidimensional understanding of cognitive effects and provide insights into the underlying mechanisms and outcomes of pharmacotherapy. The current knowledge paves the way for future research to understand better the cognitive domains and their connection with various pain dimensions to improve therapeutic management and avoid unfavorable cognitive outcomes.

## Conclusion

The past two decades have provided tremendous insights into the multifaceted role of pain in modulating cognitive domains and vice versa. This comprehensive review focused on the multidimensional role of pain in several cognitive domains including attention, memory, processing, executive functioning, decision making, psychomotor efficiency and reaction time highlights the current understanding of the intricate association between pain and cognition. We also provided insights into the role of various pharmacologic and non-pharmacologic approaches in the management of pain and the cognitive implications associated with it. It is imperative to understand the precise nature of the cognitive task affected by chronic pain. This understanding is crucial to tailor pain management therapy to the requirement of the individual. This personalized multimodal pain management allows improvement in long term quality of life and facilitates recovery.

## Author Contributions

TK and VR: involve in the planning, critical thinking, and writing the manuscript. Both authors contributed to the article and approved the submitted version.

## Conflict of Interest

The authors declare that the research was conducted in the absence of any commercial or financial relationships that could be construed as a potential conflict of interest.

## References

[B1] Al Mahrouqi M. M. MacDonald D. A. Vicenzino B. Smith M. D. (2020). Quality of life, function and disability in individuals with chronic ankle symptoms: a cross-sectional online survey. *J. Foot Ankle Res.* 13:67. 10.1186/s13047-020-00432-w 33198773 PMC7667748

[B2] Alemanno F. Houdayer E. Emedoli D. Locatelli M. Mortini P. Mandelli C. (2019). Efficacy of virtual reality to reduce chronic low back pain: proof-of-concept of a non-pharmacological approach on pain, quality of life, neuropsychological and functional outcome. *PLoS One* 14:e0216858. 10.1371/journal.pone.0216858 31120892 PMC6532874

[B3] Anand K. J. S. Willson D. F. Berger J. Harrison R. Meert K. L. Zimmerman J. (2010). Tolerance and withdrawal from prolonged opioid use in critically ill children. *Pediatrics* 125 e1208–e1225. 10.1542/peds.2009-0489 20403936 PMC3275643

[B4] Antepohl W. Kiviloog L. Andersson J. Gerdle B. (2003). Cognitive impairment in patients with chronic whiplash-associated disorder–a matched control study. *Neurorehabilitation* 18 307–315. 14757927

[B5] Apkarian V. A. Sosa Y. Krauss B. R. Thomas S. P. Fredrickson B. E. Levy R. E. (2004). Chronic pain patients are impaired on an emotional decision-making task. *Pain* 108 129–136. 10.1016/j.pain.2003.12.015 15109516

[B6] Baddeley A. (2010). Working memory. *Curr. Biol.* 20 R136–R140. 10.1016/j.cub.2009.12.014 20178752

[B7] Barnhart W. R. Buelow M. T. Trost Z. (2019). Effects of acute pain and pain-related fear on risky decision-making and effort during cognitive tests. *J. Clin. Exp. Neuropsychol.* 41 1033–1047. 10.1080/13803395.2019.1646711 31366275

[B8] Barrett D. Brintz C. E. Zaski A. M. Edlund M. J. (2020). Dialectical pain management: feasibility of a hybrid third-wave cognitive behavioral therapy approach for adults receiving opioids for chronic pain. *Pain Med.* 10.1093/pm/pnaa361 [Epub ahead of print]. 33175158 PMC8139810

[B9] Basbaum A. I. Bautista D. M. Scherrer G. Julius D. (2009). Cellular and molecular mechanisms of pain. *Cell* 139 267–284. 10.1016/j.cell.2009.09.028 19837031 PMC2852643

[B10] Benyamin R. Trescot A. M. Datta S. Buenaventura R. Adlaka R. Sehgal N. (2008). Opioid complications and side effects. *Pain Physician* 11(2 Suppl.), S105–S120. 18443635

[B11] Berryman C. Stanton T. R. Jane Bowering K. Tabor A. McFarlane A. Lorimer Moseley G. (2013). Evidence for working memory deficits in chronic pain: a systematic review and meta-analysis. *Pain* 154 1181–1196. 10.1016/j.pain.2013.03.002 23707355

[B12] Bourne S. Machado A. G. Nagel S. J. (2014). Basic anatomy and physiology of pain pathways. *Neurosurg. Clin. N. Am.* 25 629–638. 10.1016/j.nec.2014.06.001 25240653

[B13] Boyette-Davis J. A. Thompson C. D. Fuchs P. N. (2008). Alterations in attentional mechanisms in response to acute inflammatory pain and morphine administration. *Neuroscience* 151 558–563. 10.1016/j.neuroscience.2007.10.032 18065152

[B14] Brown E. N. Pavone K. J. Naranjo M. (2018). Multimodal general anesthesia: theory and practice. *Anesth. Analg.* 127 1246–1258. 10.1213/ane.0000000000003668 30252709 PMC6203428

[B15] Calandre E. P. Bembibre J. Arnedo M. L. Becerra D. (2002). Cognitive disturbances and regional cerebral blood flow abnormalities in migraine patients: their relationship with the clinical manifestations of the illness. *Cephalalgia* 22 291–302. 10.1046/j.1468-2982.2002.00370.x 12100092

[B16] Cao J. Tu Y. Orr S. P. Lang C. Park J. Vangel M. (2019). analgesic effects evoked by real and imagined acupuncture: a neuroimaging study. *Cereb. Cortex* 29 3220–3231. 10.1093/cercor/bhy190 30137262 PMC7302519

[B17] Casey K. L. Lorenz J. (2000). The determinants of pain revisited: coordinates in sensory space. *Pain Res. Manage.* 5 197–204. 10.1155/2000/586814

[B18] Ceko M. Bushnell M. C. Fitzcharles M.-A. Schweinhardt P. (2013). Fibromyalgia interacts with age to change the brain. *Neuroimage Clin.* 3 249–260. 10.1016/j.nicl.2013.08.015 24273710 PMC3814958

[B19] Cherrier M. M. Amory J. K. Ersek M. Risler L. Shen D. D. (2009). Comparative cognitive and subjective side effects of immediate-release oxycodone in healthy middle-aged and older adults. *J. Pain* 10 1038–1050. 10.1016/j.jpain.2009.03.017 19729346 PMC2757528

[B20] Chuan A. Zhou J. J. Hou R. M. Stevens C. J. Bogdanovych A. (2020). Virtual reality for acute and chronic pain management in adult patients: a narrative review. *Anaesthesia* 76 695–704. 10.1111/anae.15202 32720308

[B21] Corder G. Castro D. C. Bruchas M. R. Scherrer G. (2018). Endogenous and exogenous opioids in pain. *Annu. Rev. Neurosci.* 41 453–473. 10.1146/annurev-neuro-080317-0-61522 29852083 PMC6428583

[B22] Cousins S. Ridsdale L. Goldstein L. H. Noble A. J. Moorey S. Seed P. (2015). A pilot study of cognitive behavioural therapy and relaxation for migraine headache: a randomised controlled trial. *J. Neurol.* 262 2764–2772. 10.1007/s00415-015-7916-z 26477023 PMC4655008

[B23] Curatolo M. La Bianca G. Cosentino G. Baschi R. Salemi G. Talotta R. (2017). Motor cortex tRNS improves pain, affective and cognitive impairment in patients with fibromyalgia: preliminary results of a randomised sham-controlled trial. *Clin. Exp. Rheumatol.* 35 (Suppl. 105), 100–105. 28681715

[B24] Day M. A. Ward L. C. Ehde D. M. Thorn B. E. Burns J. Barnier A. (2019). A pilot randomized controlled trial comparing mindfulness meditation, cognitive therapy, and mindfulness-based cognitive therapy for chronic low back pain. *Pain Med.* 20 2134–2148. 10.1093/pm/pny273 30605517

[B25] Day M. A. Ward L. C. Thorn B. E. Burns J. Ehde D. M. Barnier A. J. (2020). Mechanisms of mindfulness meditation, cognitive therapy, and mindfulness-based cognitive therapy for chronic low back pain. *Clin. J. Pain* 36 740–749. 10.1097/AJP.0000000000000862 32694318

[B26] Devane W. A. Breuer A. Sheskin T. Järbe T. U. Eisen M. S. Mechoulam R. (1992). A novel probe for the cannabinoid receptor. *J. Med. Chem.* 35 2065–2069. 10.1021/jm00089a018 1317925

[B27] Dick B. D. Rashiq S. (2007). Disruption of attention and working memory traces in individuals with chronic pain. *Anesth. Analg.* 104 1223–1229. 10.1213/01.ane.0000263280.49786.f5 17456678

[B28] Doan L. Manders T. Wang J. (2015). Neuroplasticity underlying the comorbidity of pain and depression. *Neural Plast.* 2015:504691. 10.1155/2015/504691 25810926 PMC4355564

[B29] Dufton B. D. (1989). Cognitive failure and chronic pain. *Int. J. Psychiatry Med.* 19 291–297. 10.2190/jdjk-0795-5bfl-5n6k 2807747

[B30] Duric V. McCarson K. E. (2006). Persistent pain produces stress-like alterations in hippocampal neurogenesis and gene expression. *J. Pain* 7 544–555. 10.1016/j.jpain.2006.01.458 16885011

[B31] Els C. Jackson T. D. Kunyk D. Lappi V. G. Sonnenberg B. Hagtvedt R. (2017). Adverse events associated with medium- and long-term use of opioids for chronic non-cancer pain: an overview of Cochrane Reviews. *Cochrane Database Syst. Rev.* 10:CD012509. 10.1002/14651858.CD012509.pub2 29084357 PMC6485910

[B32] Emerson N. M. Meeker T. J. Greenspan J. D. Saffer M. I. Campbell C. M. Korzeniewska A. (2020). Missed targets, reaction times, and arousal are related to trait anxiety and attention to pain during an experimental vigilance task with a painful target. *J. Neurophysiol.* 123 462–472. 10.1152/jn.00331.2019 31596643 PMC7052634

[B33] Filley C. M. (2002). The Neuroanatomy of Attention. *Semin. Speech Lang.* 23 089–098. 10.1055/s-2002-24985 11951169

[B34] Gellman M. Rick Turner J. (eds) (2013). *Encyclopedia of Behavioral Medicine.* New York, NY: Springer. 10.1007/978-1-4419-1005-9

[B35] Gil-Gouveia R. Oliveira A. G. Martins I. P. (2015). Cognitive dysfunction during migraine attacks: a study on migraine without aura. *Cephalalgia* 35 662–674. 10.1177/0333102414553823 25324500

[B36] Gilliam W. P. Schumann M. E. Cunningham J. L. Evans M. M. Luedtke C. A. Morrison E. J. (2020). Pain catastrophizing as a treatment process variable in cognitive behavioral therapy for adults with chronic pain. *Eur. J. Pain* 25 339–347. 10.1002/ejp.1671 33030769

[B37] Glare P. Overton S. Aubrey K. (2020). Transition from acute to chronic pain: Where cells, systems and society meet. *Pain Manage.* 10 421–436. 10.2217/pmt-2019-0039 33111634

[B38] Gottin L. Finco G. Polati E. Bartoloni A. Zanoni L. Bianchin E. (1995). [The pre-emptive analgesia in the treatment of postoperative pain]. *Chir. Ital.* 47 12–19. 9480188

[B39] Grisart J. M. Van der Linden M. (2001). Conscious and automatic uses of memory in chronic pain patients. *Pain* 94 305–313. 10.1016/s0304-3959(01)00366-9 11731067

[B40] Gulyaeva N. V. (2017). Molecular mechanisms of neuroplasticity: an expanding universe. *Biochemistry* 82 237–242. 10.1134/S0006297917030014 28320264

[B41] Hansen G. R. Streltzer J. (2005). The psychology of pain. *Emerg. Med. Clin. North Am.* 23 339–348. 10.1016/j.emc.2004.12.005 15829386

[B42] Hart R. P. Martelli M. F. Zasler N. D. (2000). Chronic pain and neuropsychological functioning. *Neuropsychol. Rev.* 10 131–149. 10.1023/a:1009020914358 10983898

[B43] Hindmarch I. Trick L. Ridout F. (2005). A double-blind, placebo- and positive-internal-controlled (alprazolam) investigation of the cognitive and psychomotor profile of pregabalin in healthy volunteers. *Psychopharmacology* 183 133–143. 10.1007/s00213-005-0172-7 16205916

[B44] Hoegh M. Seminowicz D. A. Graven-Nielsen T. (2019). Delayed effects of attention on pain sensitivity and conditioned pain modulation. *Eur. J. Pain* 23 1850–1862. 10.1002/ejp.1458 31343803

[B45] Hu Y. Yang J. Hu Y. Wang Y. Li W. (2010). Amitriptyline rather than lornoxicam ameliorates neuropathic pain-induced deficits in abilities of spatial learning and memory. *Eur. J. Anaesthesiol.* 27 162–168. 10.1097/EJA.0b013e328331a3d5 19915478

[B46] Huang L. Dong H. J. Wang X. Wang Y. Xiao Z. (2017). Duration and frequency of migraines affect cognitive function: evidence from neuropsychological tests and event-related potentials. *J. Headache Pain* 18:54. 10.1186/s10194-017-0758-6 28477306 PMC5419957

[B47] Iezzi T. Archibald Y. Barnett P. Klinck A. Duckworth M. (1999). Neurocognitive performance and emotional status in chronic pain patients. *J. Behav. Med.* 22 205–216. 10.1023/a:1018791622441 10422614

[B48] Iezzi T. Duckworth M. P. Vuong L. N. Archibald Y. M. Klinck A. (2004). Predictors of neurocognitive performance in chronic pain patients. *Int. J. Behav. Med.* 11 56–61. 10.1207/s15327558ijbm1101_7 15194520

[B49] Institute of Medicine, and Committee on Pain, Disability, and Chronic Illness Behavior (1987). *Pain and Disability: Clinical, Behavioral, and Public Policy Perspectives*, eds Osterweis M. Kleinman A. Mechanic D. (Washington, DC: National Academies Press). 25032476

[B50] Jacobson M. L. Wulf H. A. Browne C. A. Lucki I. (2018). Opioid modulation of cognitive impairment in depression. *Prog. Brain Res.* 239 1–48. 10.1016/bs.pbr.2018.07.007 30314565 PMC6859061

[B51] Jamison R. N. Schein J. R. Vallow S. Ascher S. Vorsanger G. J. Katz N. P. (2003). Neuropsychological effects of long-term opioid use in chronic pain patients. *J. Pain Symptom Manage.* 26 913–921. 10.1016/s0885-3924(03)00310-5 14527760

[B52] Jensen M. P. Thorn B. E. Carmody J. Keefe F. J. Burns J. W. (2018). The role of cognitive content and cognitive processes in chronic pain: An important distinction? *Clin. J. Pain* 34 391–401. 10.1097/AJP.0000000000000559 28926413 PMC5876060

[B53] Ji G. Sun H. Fu Y. Li Z. Pais-Vieira M. Galhardo V. (2010). Cognitive impairment in pain through amygdala-driven prefrontal cortical deactivation. *J. Neurosci.* 30 5451–5464. 10.1523/jneurosci.0225-10.2010 20392966 PMC2868074

[B54] Johnston I. N. Maier S. F. Rudy J. W. Watkins L. R. (2012). Post-conditioning experience with acute or chronic inflammatory pain reduces contextual fear conditioning in the rat. *Behav. Brain Res.* 226 361–368. 10.1016/j.bbr.2011.08.048 21920390 PMC5652308

[B55] Joshi V. V. Patel N. D. Rehan M. A. Kuppa A. (2019). Mysterious mechanisms of memory formation: are the answers hidden in synapses? *Cureus* 11:e5795. 10.7759/cureus.5795 31728242 PMC6827877

[B56] Kikkert S. Mezue M. O’Shea J. Henderson Slater D. Johansen-Berg H. Tracey I. (2019). Neural basis of induced phantom limb pain relief. *Ann. Neurol.* 85 59–73. 10.1002/ana.25371 30383312 PMC6492189

[B57] Lai K.-L. Niddam D. M. Fuh J.-L. Chen W.-T. Wu J.-C. Wang S.-J. (2020). Cortical morphological changes in chronic migraine in a Taiwanese cohort: surface- and voxel-based analyses. *Cephalalgia* 40 575–585. 10.1177/0333102420920005 32299230

[B58] Lawlor P. G. (2002). The panorama of opioid-related cognitive dysfunction in patients with cancer: a critical literature appraisal. *Cancer* 94 1836–1853. 10.1002/cncr.10389 11920548

[B59] Lazaridou A. Kim J. Cahalan C. M. Loggia M. L. Franceschelli O. Berna C. (2017). Effects of cognitive-behavioral therapy (CBT) on brain connectivity supporting catastrophizing in fibromyalgia. *Clin. J. Pain* 33 215–221. 10.1097/AJP.0000000000000422 27518491 PMC5296218

[B60] Lee D. M. Pendleton N. Tajar A. O’Neill T. W. O’Connor D. B. Bartfai G. (2010). Chronic widespread pain is associated with slower cognitive processing speed in middle-aged and older European men. *Pain* 151 30–36. 10.1016/j.pain.2010.04.024 20646831

[B61] Lee S. J. Bora S. Austin N. C. Westerman A. Henderson J. M. T. (2020). Neurodevelopmental outcomes of children born to opioid-dependent mothers: a systematic review and meta-analysis. *Acad. Pediatr.* 20 308–318. 10.1016/j.acap.2019.11.005 31734383

[B62] Legrain V. Van Damme S. Eccleston C. Davis K. D. Seminowicz D. A. Crombez G. (2009). A neurocognitive model of attention to pain: behavioral and neuroimaging evidence. *Pain* 144 230–232. 10.1016/j.pain.2009.03.020 19376654

[B63] Lin J. C. Chu L. F. Stringer E. A. Baker K. S. Sayyid Z. N. Sun J. (2016). One Month of Oral Morphine Decreases Gray Matter Volume in the Right Amygdala of Individuals with Low Back Pain: confirmation of Previously Reported Magnetic Resonance Imaging Results. *Pain. Med.* 17 1497–1504. 10.1093/pm/pnv047 26814280 PMC4921346

[B64] Lindner M. D. Plone M. A. Francis J. M. Cain C. K. (1999). Chronic morphine reduces pain-related disability in a rodent model of chronic, inflammatory pain. *Exp. Clin. Psychopharmacol.* 7 187–197. 10.1037/1064-1297.7.3.187 10472506

[B65] Lindner S. Latoschik M.-E. Rittner H. (2020). [Use of Virtual Reality as a Component of Acute and Chronic Pain Treatment]. *Anasthesiol. Intensivmed. Notfallmed. Schmerzther.* 55 549–561. 10.1055/a-1022-3038 32916738

[B66] Martinsen S. Flodin P. Berrebi J. Löfgren M. Bileviciute-Ljungar I. Mannerkorpi K. (2018). The role of long-term physical exercise on performance and brain activation during the Stroop colour word task in fibromyalgia patients. *Clin. Physiol. Funct. Imaging* 38 508–516. 10.1111/cpf.12449 28627125

[B67] McCracken L. M. Iverson G. L. (2001). Predicting complaints of impaired cognitive functioning in patients with chronic pain. *J. Pain Symptom Manage.* 21 392–396. 10.1016/s0885-3924(01)00267-6 11369160

[B68] McGuire B. E. (2013). Chronic pain and cognitive function. *Pain* 154 964–965. 10.1016/j.pain.2013.04.008 23659915

[B69] Melzack R. Coderre T. J. Katz J. Vaccarino A. L. (2006). Central neuroplasticity and pathological pain. *Ann. N.Y. Acad. Sci.* 933 157–174. 10.1111/j.1749-6632.2001.tb05822.x 12000018

[B70] Millecamps M. Etienne M. Jourdan D. Eschalier A. Ardid D. (2004). Decrease in non-selective, non-sustained attention induced by a chronic visceral inflammatory state as a new pain evaluation in rats. *Pain* 109 214–224. 10.1016/j.pain.2003.12.028 15157681

[B71] Minkova L. Habich A. Peter J. Kaller C. P. Eickhoff S. B. Klöppel S. (2017). Gray matter asymmetries in aging and neurodegeneration: a review and meta-analysis. *Hum. Brain Mapp.* 38 5890–5904. 28856766 10.1002/hbm.23772 PMC6866813

[B72] Mirsky A. F. Anthony B. J. Duncan C. C. Ahearn M. B. Kellam S. G. (1991). Analysis of the elements of attention: a neuropsychological approach. *Neuropsychol. Rev.* 2 109–145. 10.1007/BF01109051 1844706

[B73] Moore D. J. Meints S. M. Lazaridou A. Johnson D. Franceschelli O. Cornelius M. (2019). the effect of induced and chronic pain on attention. *J. Pain* 20 1353–1361. 10.1016/j.jpain.2019.05.004 31077797

[B74] Moriarty O. McGuire B. E. Finn D. P. (2011). The effect of pain on cognitive function: a review of clinical and preclinical research. *Prog. Neurobiol.* 93 385–404. 10.1016/j.pneurobio.2011.01.002 21216272

[B75] Müller H. (2000). [Neuroplasticity and chronic pain]. *Anasthesiol. Intensivmed. Notfallmed. Schmerzther.* 35 274–284. 10.1055/s-2000-352 10858836

[B76] Muñoz M. Esteve R. (2005). Reports of memory functioning by patients with chronic pain. *Clin. J. Pain* 21 287–291. 10.1097/01.ajp.0000173993.53733.2e 15951644

[B77] Mutso A. A. Radzicki D. Baliki M. N. Huang L. Banisadr G. Centeno M. V. (2012). Abnormalities in hippocampal functioning with persistent pain. *J. Neurosci.* 32 5747–5756. 10.1523/JNEUROSCI.0587-12.2012 22539837 PMC3365570

[B78] Nadar M. S. Jasem Z. Manee F. S. (2016). The cognitive functions in adults with chronic pain: a comparative study. *Pain Res. Manage.* 2016 5719380. 10.1155/2016/5719380 28127233 PMC5227177

[B79] Neugebauer V. (2015). Amygdala pain mechanisms. *Handb. Exp. Pharmacol.* 227 261–284. 10.1007/978-3-662-46450-2_13 25846623 PMC4701385

[B80] Neugebauer V. Li W. Bird G. C. Han J. S. (2004). The amygdala and persistent pain. *Neuroscientist* 10 221–234. 10.1177/1073858403261077 15155061

[B81] Neumann S. Doubell T. P. Leslie T. Woolf C. J. (1996). Inflammatory pain hypersensitivity mediated by phenotypic switch in myelinated primary sensory neurons. *Nature* 384 360–364. 10.1038/384360a0 8934522

[B82] Oláh C. Schwartz N. Denton C. Kardos Z. Putterman C. Szekanecz Z. (2020). Cognitive dysfunction in autoimmune rheumatic diseases. *Arthritis Res. Ther.* 22:78. 10.1186/s13075-020-02180-5 32293528 PMC7158026

[B83] Pais-Vieira M. Lima D. Galhardo V. (2009). Sustained attention deficits in rats with chronic inflammatory pain. *Neurosci. Lett.* 463 98–102. 10.1016/j.neulet.2009.07.050 19631256

[B84] Pask S. Dell’Olio M. Murtagh F. E. M. Boland J. W. (2020). The effects of opioids on cognition in older adults with cancer and chronic noncancer pain: a systematic review. *J. Pain Symptom Manage* 59 871–893.e1. 10.1016/j.jpainsymman.2019.10.022 31678462

[B85] Petrovic P. Petersson K. M. Ghatan P. H. Stone-Elander S. Ingvar M. (2000). Pain-related cerebral activation is altered by a distracting cognitive task. *Pain* 85 19–30. 10.1016/s0304-3959(99)00232-8 10692599

[B86] Planchuelo-Gómez Á. García-Azorín D. Guerrero Á. L. Rodríguez M. Aja-Fernández S. de Luis-García R. (2020). Gray matter structural alterations in chronic and episodic migraine: a morphometric magnetic resonance imaging study. *Pain Med.* 21 2997–3011. 10.1093/pm/pnaa271 33040149

[B87] Portenoy R. K. (2011). Treatment of cancer pain. *Lancet* 377 2236–2247. 10.1016/S0140-6736(11)60236-5 21704873

[B88] Pourmand A. Davis S. Marchak A. Whiteside T. Sikka N. (2018). Virtual reality as a clinical tool for pain management. *Curr. Pain Headache Rep.* 22:53. 10.1007/s11916-018-0708-2 29904806

[B89] Povedano M. Gascón J. Gálvez R. Ruiz M. Rejas J. (2007). Cognitive function impairment in patients with neuropathic pain under standard conditions of care. *J. Pain Symptom Manage.* 33 78–89. 10.1016/j.jpainsymman.2006.07.012 17196909

[B90] Pozeg P. Palluel E. Ronchi R. Solcà M. Al-Khodairy A.-W. Jordan X. (2017). Virtual reality improves embodiment and neuropathic pain caused by spinal cord injury. *Neurology* 89 1894–1903. 10.1212/WNL.0000000000004585 28986411 PMC5664293

[B91] Qian Y. Xia T. Cui Y. Chu S. Ma Z. Gu X. (2019). The role of CaMKII in neuropathic pain and fear memory in chronic constriction injury in rats. *Int. J. Neurosci.* 129 146–154. 10.1080/00207454.2018.1512986 30118368

[B92] Raja S. N. Carr D. B. Cohen M. Finnerup N. B. Flor H. Gibson S. (2020). The revised International Association for the Study of Pain definition of pain: concepts, challenges, and compromises. *Pain* 161 1976–1982. 10.1097/j.pain.0000000000001939 32694387 PMC7680716

[B93] Ramachandran V. S. Rogers-Ramachandran D. (2000). Phantom limbs and neural plasticity. *Arch. Neurol.* 57 317–320. 10.1001/archneur.57.3.317 10714655

[B94] Rischer K. M. González-Roldán A. M. Montoya P. Gigl S. Anton F. van der Meulen M. (2020). Distraction from pain: the role of selective attention and pain catastrophizing. *Eur. J. Pain* 24 1880–1891. 10.1002/ejp.1634 32677265 PMC7689692

[B95] Rodríguez-Andreu J. Ibáñez-Bosch R. Portero-Vázquez A. Masramon X. Rejas J. Gálvez R. (2009). Cognitive impairment in patients with fibromyalgia syndrome as assessed by the mini-mental state examination. *BMC Musculoskelet. Disord.* 10:162. 10.1186/1471-2474-10-162 20025750 PMC2811106

[B96] Roozendaal B. Okuda S. de Quervain D. J.-F. McGaugh J. L. (2006). Glucocorticoids interact with emotion-induced noradrenergic activation in influencing different memory functions. *Neuroscience* 138 901–910. 10.1016/j.neuroscience.2005.07.049 16310958

[B97] Ryan C. M. (2005). Diabetes, aging, and cognitive decline. *Neurobiol. Aging* 26 (Suppl. 1), 21–25. 10.1016/j.neurobiolaging.2005.09.006 16213627

[B98] Ryan C. M. Williams T. M. Finegold D. N. Orchard T. J. (1993). Cognitive dysfunction in adults with type 1 (insulin-dependent) diabetes mellitus of long duration: effects of recurrent hypoglycaemia and other chronic complications. *Diabetologia* 36 329–334. 10.1007/BF00400236 8477878

[B99] Sadhasivam S. Alankar S. Maturi R. Vishnubhotla R. V. Mudigonda M. Pawale D. (2020). Inner engineering practices and advanced 4-day Isha yoga retreat are associated with cannabimimetic effects with increased endocannabinoids and short-term and sustained improvement in mental health: a prospective observational study of meditators. *Evid. Based Complement. Alternat. Med.* 2020:8438272. 10.1155/2020/8438272 32595741 PMC7293737

[B100] Said F. A. Betoni T. B. Magalhaes V. Nisihara R. Skare T. L. (2019). Rheumatoid arthritis and cognition dysfunction: lack of association with cumulative glucocorticoid use. *Immunopharmacol. Immunotoxicol.* 41 565–567. 10.1080/08923973.2019.1679170 31625439

[B101] Salinsky M. Storzbach D. Munoz S. (2010). Cognitive effects of pregabalin in healthy volunteers: a double-blind, placebo-controlled trial. *Neurology* 74 755–761. 10.1212/WNL.0b013e3181d25b34 20194915

[B102] Sawangjit A. Oyanedel C. N. Niethard N. Salazar C. Born J. Inostroza M. (2018). The hippocampus is crucial for forming non-hippocampal long-term memory during sleep. *Nature* 564 109–113. 10.1038/s41586-018-0716-8 30429612

[B103] Shannon H. E. Love P. L. (2004). Effects of antiepileptic drugs on working memory as assessed by spatial alternation performance in rats. *Epilepsy Behav.* 5 857–865. 10.1016/j.yebeh.2004.08.017 15582833

[B104] Shannon H. E. Love P. L. (2005). Effects of antiepileptic drugs on attention as assessed by a five-choice serial reaction time task in rats. *Epilepsy Behav.* 7 620–628. 10.1016/j.yebeh.2005.08.017 16253568

[B105] Shiffrin R. M. (1977). “Commentary on “human memory: a proposed system and its control processes,” in *Human Memory*, eds C. Atkinson Richard and M. Shiffrin Richard (Amsterdam: Elsevier), 1–5. 10.1016/b978-0-12-121050-2.50005-3

[B106] Shuchang H. Mingwei H. Hongxiao J. Si W. Xing Y. Antonius D. (2011). Emotional and neurobehavioural status in chronic pain patients. *Pain Res. Manage.* 16 41–43. 10.1155/2011/825636 21369540 PMC3052406

[B107] Sjøgren P. Christrup L. L. Petersen M. A. Højsted J. (2005). Neuropsychological assessment of chronic non-malignant pain patients treated in a multidisciplinary pain centre. *Eur. J. Pain* 9 453–462. 10.1016/j.ejpain.2004.10.005 15979026

[B108] Sloan P. Hollins M. (2017). Attention and pain: are auditory distractors special? *Exp. Brain Res.* 235 1593–1602. 10.1007/s00221-017-4903-x 28260156

[B109] Stella N. Schweitzer P. Piomelli D. (1997). A second endogenous cannabinoid that modulates long-term potentiation. *Nature* 388 773–778. 10.1038/42015 9285589

[B110] Tajerian M. Hung V. Nguyen H. Lee G. Joubert L.-M. Malkovskiy A. V. (2018). The hippocampal extracellular matrix regulates pain and memory after injury. *Mol. Psychiatry* 23 2302–2313. 10.1038/s41380-018-0209-z 30254235 PMC6294737

[B111] Tassain V. Attal N. Fletcher D. Brasseur L. Dégieux P. Chauvin M. (2003). Long term effects of oral sustained release morphine on neuropsychological performance in patients with chronic non-cancer pain. *Pain* 104 389–400. 10.1016/s0304-3959(03)00047-2 12855350

[B112] Thompson J. M. Neugebauer V. (2017). Amygdala plasticity and pain. *Pain Res. Manage.* 2017:8296501. 10.1155/2017/8296501 29302197 PMC5742506

[B113] Thorp S. L. Suchy T. Vadivelu N. Helander E. M. Urman R. D. Kaye A. D. (2018). Functional connectivity alterations: novel therapy and future implications in chronic pain management. *Pain Physician* 21 E207–E214. 29871376

[B114] Treede R.-D. Rief W. Barke A. Aziz Q. Bennett M. I. Benoliel R. (2019). Chronic pain as a symptom or a disease: the IASP Classification of Chronic Pain for the International Classification of Diseases (ICD-11). *Pain* 160 19–27. 10.1097/j.pain.0000000000001384 30586067

[B115] Tseng M.-T. Kong Y. Eippert F. Tracey I. (2017). Determining the neural substrate for encoding a memory of human pain and the influence of anxiety. *J. Neurosci.* 37 11806–11817. 10.1523/JNEUROSCI.0750-17.2017 29097595 PMC5719969

[B116] Tyng C. M. Amin H. U. Saad M. N. M. Malik A. S. (2017). The influences of emotion on learning and memory. *Front. Psychol.* 8:1454. 10.3389/fpsyg.2017.01454 28883804 PMC5573739

[B117] Valet M. (2004). Distraction modulates connectivity of the cingulo-frontal cortex and the midbrain during pain? An fMRI analysis. *Pain* 109 399–408. 10.1016/s0304-3959(04)00095-8 15157701

[B118] Valet M. Sprenger T. Boecker H. Willoch F. Rummeny E. Conrad B. (2004). Distraction modulates connectivity of the cingulo-frontal cortex and the midbrain during pain—an fMRI analysis. *Pain* 109 399–408. 10.1016/j.pain.2004.02.033 15157701

[B119] van der Leeuw G. Leveille S. G. Dong Z. Shi L. Habtemariam D. Milberg W. (2018). Chronic Pain and Attention in Older Community-Dwelling Adults. *J. Am. Geriatr. Soc.* 66 1318–1324. 10.1111/jgs.15413 29876923 PMC6181226

[B120] Vekhter D. Robbins M. S. Minen M. Buse D. C. (2020). Efficacy and feasibility of behavioral treatments for migraine, headache, and pain in the acute care setting. *Curr. Pain Headache Rep.* 24:66. 10.1007/s11916-020-00899-z 32979092 PMC7754250

[B121] Verdejo-García A. Bechara A. Recknor E. C. Pérez-García M. (2006). Executive dysfunction in substance dependent individuals during drug use and abstinence: an examination of the behavioral, cognitive and emotional correlates of addiction. *J. Int. Neuropsychol. Soc.* 12 405–415. 10.1017/s1355617706060486 16903133

[B122] Verdejo-García A. López-Torrecillas F. Calandre E. P. Delgado-Rodríguez A. Bechara A. (2009). Executive function and decision-making in women with fibromyalgia. *Arch. Clin. Neuropsychol.* 24 113–122. 10.1093/arclin/acp014 19395361

[B123] Villemure C. Bushnell M. C. (2002). Cognitive modulation of pain: how do attention and emotion influence pain processing? *Pain* 95 195–199. 10.1016/s0304-3959(02)00007-6 11839418

[B124] Wager T. D. Atlas L. Y. Lindquist M. A. Roy M. Woo C.-W. Kross E. (2013). An fMRI-based neurologic signature of physical pain. *N. Engl. J. Med.* 368 1388–1397. 10.1056/NEJMoa1204471 23574118 PMC3691100

[B125] Walankar P. P. Panhale V. P. Patil M. M. (2020). Psychosocial factors, disability and quality of life in chronic shoulder pain patients with central sensitization. *Health Psychol. Res.* 8:8874. 10.4081/hpr.2020.8874 33123644 PMC7588851

[B126] Wallace M. S. Marcotte T. D. Umlauf A. Gouaux B. Atkinson J. H. (2015). Efficacy of inhaled cannabis on painful diabetic neuropathy. *J. Pain* 16 616–627. 10.1016/j.jpain.2015.03.008 25843054 PMC5152762

[B127] Weiner D. K. Rudy T. E. Morrow L. Slaboda J. Lieber S. (2006). The relationship between pain, neuropsychological performance, and physical function in community-dwelling older adults with chronic low back pain. *Pain. Med.* 7 60–70. 10.1111/j.1526-4637.2006.00091.x 16533199

[B128] Wilder-Smith O. H. (1995). [Pre-emptive analgesia]. *Anaesthesist* 44 (Suppl. 3), S529–S534. 8592963

[B129] Wildes M. Bigand T. L. Layton M. E. Wilson M. (2020). Cannabis use and cognition in adults prescribed opioids for persistent pain. *Pain Manage. Nurs.* 21 94–99. 10.1016/j.pmn.2019.06.014 31405787

[B130] Woodhams S. G. Chapman V. Finn D. P. Hohmann A. G. Neugebauer V. (2017). The cannabinoid system and pain. *Neuropharmacology* 124 105–120. 10.1016/j.neuropharm.2017.06.015 28625720 PMC5785108

[B131] Xie Y.-F. Huo F.-Q. Tang J.-S. (2009). Cerebral cortex modulation of pain. *Acta Pharmacol. Sin.* 30 31–41. 10.1038/aps.2008.14 19079295 PMC4006538

[B132] Yam M. Loh Y. Tan C. Adam S. K. Manan N. A. Basir R. (2018). General pathways of pain sensation and the major neurotransmitters involved in pain regulation. *Int. J. Mol. Sci.* 19:2164. 10.3390/ijms19082164 30042373 PMC6121522

[B133] Zaletel I. Filipović D. Puškaš N. (2016). Chronic stress, hippocampus and parvalbumin-positive interneurons: What do we know so far? *Rev. Neurosci.* 27 397–409. 26751865 10.1515/revneuro-2015-0042

[B134] Zetterqvist V. Gentili C. Rickardsson J. Sörensen I. Wicksell R. K. (2020). Internet-delivered acceptance and commitment therapy for adolescents with chronic pain and their parents: a nonrandomized pilot trial. *J. Pediatr. Psychol.* 45 990–1004. 10.1093/jpepsy/jsaa060 32974656

[B135] Zgierska A. E. Burzinski C. A. Cox J. Kloke J. Stegner A. Cook D. B. (2016). Mindfulness meditation and cognitive behavioral therapy intervention reduces pain severity and sensitivity in opioid-treated chronic low back pain: pilot findings from a randomized controlled trial. *Pain Med.* 17 1865–1881. 10.1093/pm/pnw006 26968850 PMC5063022

